# Rhinovirus: A Narrative Review on Its Genetic Characteristics, Pediatric Clinical Presentations, and Pathogenesis

**DOI:** 10.3389/fped.2021.643219

**Published:** 2021-03-22

**Authors:** Etienne Bizot, Anais Bousquet, Maelle Charpié, Florence Coquelin, Servane Lefevre, Justin Le Lorier, Margaux Patin, Perrine Sée, Eytan Sarfati, Servane Walle, Benoit Visseaux, Romain Basmaci

**Affiliations:** ^1^Department of Microbiology, Robert Debré Hospital, APHP, Paris, France; ^2^Departement of Emergency and Pediatrics, Louis-Mourier Hospital, APHP, Colombes, France; ^3^Department of Virology, Bichat Hospital, APHP, Paris, France; ^4^University of Paris, Inserm, IAME, UMR1137, Paris, France

**Keywords:** rhinovirus, respiratory virus, children, enterovirus, respiratory tract infection

## Abstract

Human rhinoviruses (HRVs) are the leading cause of common colds. With the development of new molecular methods since the 2000s, HRVs have been increasingly involved among severe clinical infections. Recent knowledge of the HRV genetic characteristics has also improved the understanding of their pathogenesis. This narrative review aims to provide a current comprehensive knowledge about this virus in the pediatric community. HRVs represent a main cause of upper and lower respiratory tract infections in children. HRV is the second virus involved in bronchiolitis and pneumonia in children, and HRV bronchiolitis has a higher risk of recurrent wheezing episode or asthma. Some recent findings described HRVs in stools, blood, or cerebrospinal fluid, thanks to new molecular techniques such as polymerase chain reaction (PCR) by detecting HRVs with high sensibility. However, the high rate of asymptomatic carriage and the prolonged excretion in postsymptomatic patients complicate interpretation. No sufficient data exist to avoid antibiotic therapy in pediatric high-risk population with HRV detection. Severe clinical presentations due to HRVs can be more frequent in specific population with chronic pathology or genetic particularity. Inflammatory response is mediated by the nuclear factor (NF)-kappa B pathway and production of interferon (IFN)-beta and IFN-gamma, interleukin 8 (IL8), and IL1b. No specific treatment or antiviral therapy exists, although research is still ongoing. Nowadays, in addition to benign diseases, HRVs are recognized to be involved in some severe clinical presentations. Recent advances in genetic knowledge or specific inflammatory response may lead to specific treatment.

## Introduction

Discovered in the 1950s, human rhinoviruses (HRVs) are mostly known as the leading cause of the “common cold”; they are ubiquitous, and HRV infections occur year round (1).

With the development of new molecular methods since the 2000s, HRVs have been increasingly involved in more severe clinical infections. Recent knowledge of the HRV genetic characteristics has also improved the understanding of their pathogenesis. This led to a large increase in publications in the recent years. In this review, we aimed to provide a comprehensive assessment of the current knowledge on HRV infections.

## Methods

We performed a narrative review on HRV describing its genetic characteristics, the current diagnostic methods, the most common pediatric clinical manifestations, and their pathogenesis.

A literature search with different free terms related to human rhinovirus in children (<18 years old) was conducted using the PubMed database until March 31, 2019. Search strategy was restricted to articles published in English and French. Potential articles were screened by title and abstract, and if relevant, the full text was assessed.

## Genetic Characteristics and Phylogeny

**Figure 1:**
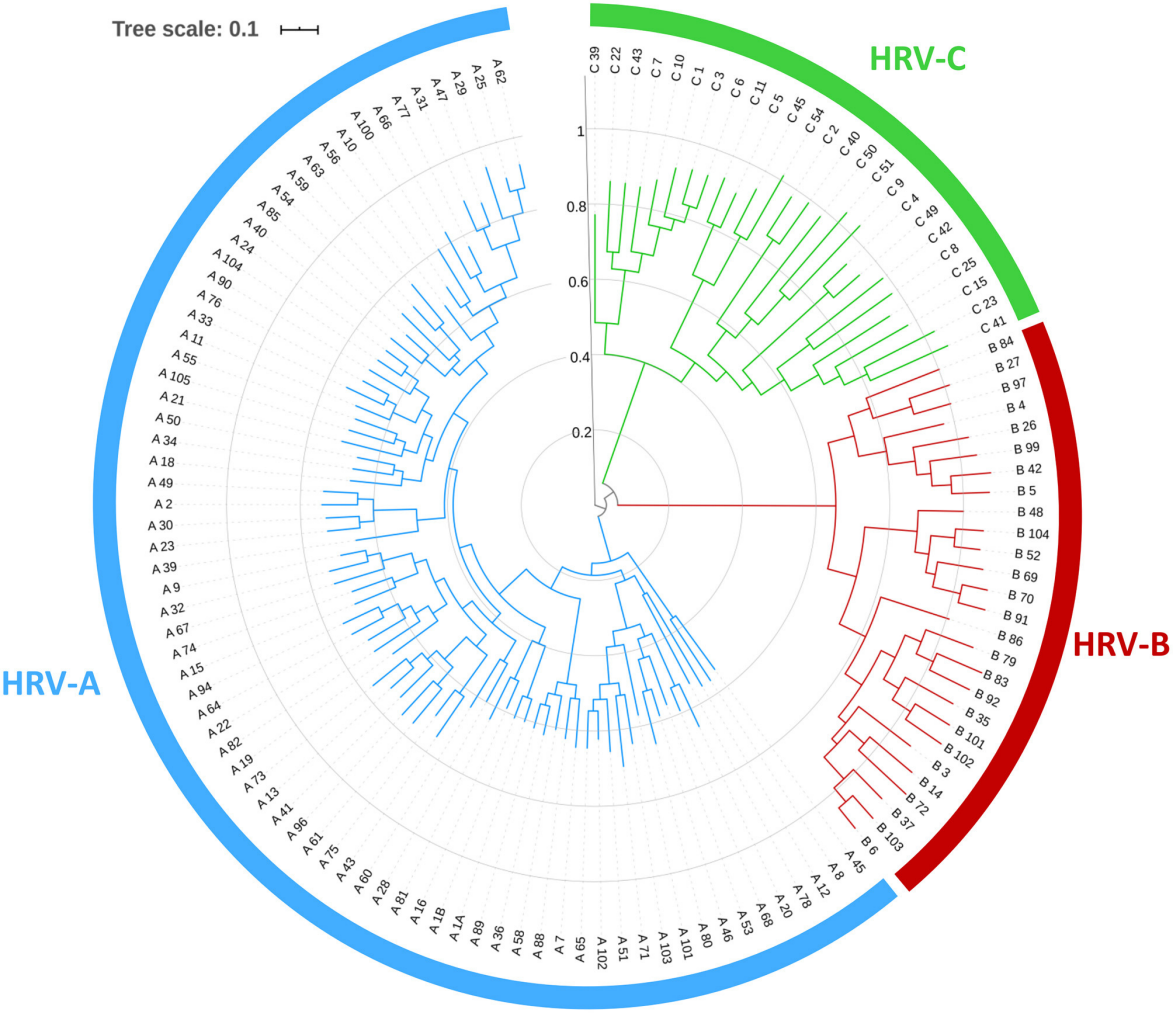
Phylogenetic tree of human rhinoviruses obtained by comparison of near full-length genome using approximated maximum of likelihood with FastTree2.1.

HRV is a member of the *Picornaviridae* family including nine genera, six of which are pathogenic for humans: *enterovirus, rhinovirus, hepatovirus, parechovirus, cardiovirus*, and *kobuvirus*. HRV is often classified into three different species, HRV-A, HRV-B, and HRV-C (Figure 1). HRV-A and HRV-B were initially described in the early 1990s, whereas HRV-C, discovered in 2003, was included in the International Committee on Taxonomy of Viruses in 2009 (2).

HRVs are non-enveloped viruses measuring 30 nm. The genome is a positive single-stranded RNA (ssRNA) of 7,200 bp. It consists of a single gene encoding 11 proteins. Four proteins (VP1, VP2, VP3, and VP4) make up the viral capsid that encases the ssRNA genome. VP1, VP2, and VP3 proteins account for the virus' antigenic diversity, while VP4 anchors the ssRNA core to the capsid. There are 60 copies of each capsid proteins, giving to the virion an icosahedral structure with a canyon in VP1 that serves as the binding site to cell surface receptors (3).

## Diagnostic Methods

### Sample Collection

HRVs are mostly detected within the first 6 days after the symptoms onset (4). In case of upper respiratory tract infections (URTIs), nasopharyngeal swabbing or aspiration appears to be more sensitive than oropharyngeal swabbing (5). In case of lower respiratory tract infections (LRTIs), samples can be obtained by tracheal or bronchial aspiration, broncho-alveolar lavage, or, more rarely, by pulmonary biopsies.

Samples should be sent in a few hours to the laboratory or kept at +4°C in a viral transport medium and sent within a few days.

#### Molecular Methods

Viral culture had low sensitivity and a long delay, up to 14 days. In 1988, Gama et al. described the first PCR-based assay able to detect HRV in primary respiratory samples (6). Real-time PCR methods and multiplex PCR (mPCR) have been recently developed and can allow the detection of HRV within 1 h with a high detection rate. These mPCR tests are expensive, ranging up to 120 € (140 USD) for some of them. Most of these assays cannot differentiate enteroviruses and HRVs as targeting the highly conserved 5′ untranslated region (UTR) region. Thus, during severe central nervous system involvement, more specific tests are required to correctly identify enteroviral infections due to D68, A71, or other enteroviruses (7, 8).

### Profit–Risk Balance

Transition to testing with an mPCR led to significantly increased detection of respiratory viruses. Thus, molecular techniques may contribute to de-escalation of antibiotics and reduction in ancillary testing, thereby offsetting the cost of the more expensive PCR test.

However, a first controlled clinical trial using rapid mPCR failed to demonstrate decreased hospital admissions, shorter length of stay, or decreased antimicrobial use for children with acute respiratory infections (9).

Thus, the cost–benefit balance of such test in children populations remains to be determined with the use of appropriate algorithms that still need to be established.

## Clinical Presentations

### Transmission and Epidemiology

HRV replicates in nasal and posterior nasopharynx mucosa (5). Transmission is mainly attributed to hand contact between persons or through fomites (10). Oral or aerosol transmission seems to be rare and dependent on viral particles concentration in the droplets. Viral loads in saliva are about 30 times lower than in nasal secretions (10).

Morikawa et al. (11), by studying seasonal fluctuations of 16 community respiratory viruses in children both symptomatic and non-symptomatic, demonstrated that HRV-A was present all year round, while HRV-C was more common in winter, and HRV-B had an unclear seasonality.

### Common Clinical Presentations

#### Asymptomatic Carriage

Asymptomatic HRV carriage is most often defined as the detection of HRV in nasal sample of children without respiratory symptoms. However, while some authors distinguished asymptomatic from prolonged carriage when children had symptoms during the previous weeks, some others did not search for this information.

It has been estimated to be 14–22% (12, 13), but the methodology was heterogeneous across studies. Thus, HRV asymptomatic carriage is difficult to confirm, as clearance from nasal mucus can occur up to 5 or 6 weeks after the onset of respiratory symptoms in children and can be even longer in case of severe immunosuppression.

#### Common Cold

Adults are considered to have an average of 2–5 episodes of common cold per year, while 7–10 episodes per year occur in children (14). Van Beten et al. found HRV in 27% of common cold episodes in 6-month-old children and 60% in 24-month-old children (12).

Although it is a benign disease, common cold is associated with significant costs in terms of medical visits, absenteeism, medical prescriptions, and inadequate use of antibiotics (15).

#### Acute Otitis Media

Acute otitis media (AOM) are often bacterial or related to bacterial and viral coinfection, but single viral infections may also be involved (16). Among 362 <1-year-old infants who underwent monthly respiratory sampling during 9 months, HRV and respiratory syncytial virus (RSV) were specifically associated with AOM occurrence (*P* < 0.001) (17).

#### Bronchiolitis

Bronchiolitis is the main cause of hospitalization in infants. Behind RSV, HRV is the second virus involved in bronchiolitis, accounting for up to 25% of cases (18).

In different studies, a shorter duration of hospitalization for HRV bronchiolitis was observed compared with RSV (19), while it has not yet been demonstrated whether a specific virus was responsible for more severe infections (20). Moreover, there is no significant difference between the prevalence of RSV and HRV among children hospitalized in pediatric intensive care units and those hospitalized on general pediatric units (21).

However, HRV seems to be more involved than RSV in infants with a high predisposition to atopy due to a higher Th2 immune response (22). Thus, HRV-related bronchiolitis leads to recurrent wheezing episodes after the first bronchiolitis in atopic children (23).

#### Pneumonia

In a large study assessing incidence and pathogen distributions of community acquired pneumonia in hospitalized children, viruses (one or more), bacteria, or viral–bacterial coinfections were identified in 66, 8, and 7% of cases, respectively (24). The most commonly detected pathogens were RSV (28%) and HRV (27%). All other viruses or bacteria were under 10%. HRV was mostly found among children aged from 5 to 17 years. However, the presence of HRV in the nasopharynx does not necessarily demonstrate its causative role in pneumonia; it can also only indicate a coincidental respiratory infection or prolonged excretion. Moreover, in this study, HRV was detected in 17% of controls, while any other virus was identified in <3% (24).

#### Asthma

Viral respiratory infections are the leading cause of asthmatic exacerbation in childhood (25). In 2018, in a large study among 958 children from 1 to 17 years old with moderate to severe asthma exacerbation, 62% were positive for at least one pathogen, with a large predominance of HRV (29%) (26).

Children hospitalized with HRV bronchiolitis had a higher risk of recurrent wheezing episode (23) and a higher risk of asthma persistence at 6 years old [odds ratio (OR), 9.8 (4.3–22.0)] (27) by contributing to the initiation and progression of airway remodeling. However, repeated HRV infections are common in infants, and only few of them developed asthma. Thus, additional risk factors such as host genetic susceptibility and key environmental exposures are also involved in asthma development. Chromosome 17q21 variants (ORMDL3 and GSDMB) have especially been shown to be associated with HRV-induced asthma in children (28).

### Specific Clinical Presentations

#### Non-respiratory Infections

HRV has been also investigated outside the respiratory sphere.

HRV was identified in about 10% of stool samples of children hospitalized for gastroenteritis (29). In 2015, HRV-C was detected after autopsy in the blood, lungs, stool, and cerebrospinal fluid (CSF) from a healthy 19-month-old patient admitted to intensive care for acute severe respiratory distress (30), highlighting potential circulation of HRV in severe pictures. Such findings still need larger investigations and confirmations in the future.

#### Infants Younger Than 3 Months Old

The management of febrile infants younger than 3 months old requires rapid medical assessment, bacteriological sampling, and frequent empirical antibiotic therapy because of frequent bacterial infections (around 10%).

A study published in 2018 (31) on febrile infants from 1 to 90 days old (*n* = 4,037) detected viral infection in 55% of cases. Among them, HRV was the most common virus detected (69%). Children being infected by a respiratory virus were less frequently infected with bacteria (6 vs. 13%). However, a bacterial infection was more frequently observed in infants with HRV than those with non-HRV viral infection [7.5 vs. 3.5%, respectively; RR = 2.12 (1.43–3.15)]. Finally, among infants aged from 1 to 28 days old, urinary tract infection, bloodstream infection, and meningitis were not significantly reduced when HRV was detected. Thus, HRV detection should not discourage antibiotic use on such young children.

#### Viral Coinfections

It is common to detect several viruses simultaneously in nasopharyngeal secretions in children. HRV and eight other viruses were searched for in 434 >5-year-old children with respiratory infection symptoms. Among them, 181 (42%) were HRV positive, 41% of which had coinfections (32). RSV was the most common virus found concomitantly with HRV. HRV nasopharyngeal aspirates viral load was lower among RSV–HRV coinfections compared with HRV monoinfections, but the duration of symptoms was longer in such coinfections than in RSV monoinfections (33). Conflicting results still exist about the causal role of HRV in coinfections, and some studies suggested that HRV inhibits superinfection by other viruses (34).

## Pathogenesis

### Viral Replication

In the upper respiratory tract, HRV replication occurs in the nasal mucosa and posterior nasopharynx, mainly in ciliated epithelial cells.

In the lower respiratory tract, HRV can be detected in bronchial biopsy, mainly in columnar epithelial cells, to a lesser extent in the basal cell layer, but also in type II pneumocytes (35).

#### Binding and Internalization

**Figure 2:**
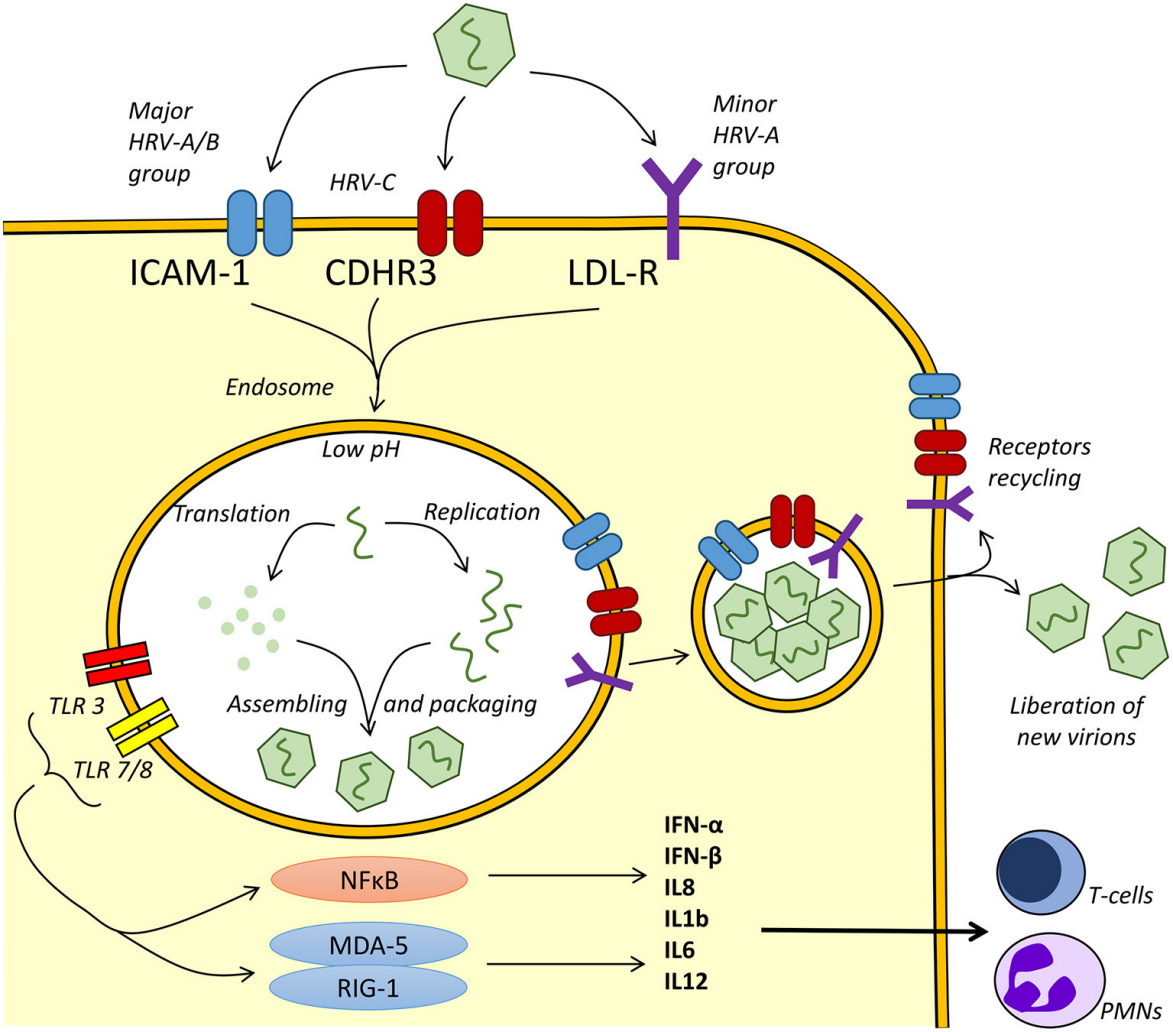
Human rhinoviruse (HRV) replication and inflammatory response. Depending on the serotype, HRVs use intercellular adhesion molecule 1 (ICAM-1), cadherin-related family 3 (CDHR3), or low-density lipoprotein receptor (LDL-R) for endocytosis. After binding, HRV upregulates ICAM-1 and other receptors expression (34). A pH drop leads to viral uncoating after the loss of the capsid protein VP4 and externalization of hydrophobic N-terminal of VP1 (36). In the endosome, viral RNAs are recognized by Toll-like receptor 3 (TLR3) and TLR7/8, while receptors are recycled to apical plasma membrane (37). Nuclear factor (NF)-kappa B pathway and the pattern recognition receptors ([RIG-1] and [MDA-5]) in the intracellular compartment are activated, leading to the production of interleukin 8 (IL8), IL1b, IL6, IL12, and interferon (IFN) beta and gamma. The cascade leads in fine to the increased production of T cells and neutrophil cytokines.

After binding to its specific receptor, thanks to canyon in VP1, HRV internalization occurs via clathrin-dependent or clathrin-independent endocytosis or via micropinocytosis (3) (Figure 2).

Intercellular adhesion molecule 1 (ICAM-1) is the epithelial receptor for most HRV-A and HRV-B serotypes, whereas the low-density lipoprotein receptor (LDL-R) is recognized by few HRV-A. HRV-C uses cadherin-related family 3 (CDHR3) for binding and replication (38).

HRV binding upregulates ICAM-1 expression at the cell surface, allowing multiple ICAM-1 molecules binding by single viral particle. This binding is required to initiate internalization and participates with the low pH environment in endosomes to the uncoating mechanism of the virions (39, 40). Then, HRV pathogen-associated molecular patterns (PAMPs) are recognized by the host cell via interaction with Toll-like receptors (TLRs).

In the endosome, viral recognition by the host pattern recognition receptors (PRRs) will trigger host defenses and induce the production of inflammatory mediators and interferons (41). Adhesion to TLR3 and TLR7/8 leads to the activation of the nuclear factor (NF)-kappa B pathway, triggering production of interferon (IFN)-beta and IFN-gamma, interleukin 8 (IL8), and IL1b leading to antiviral and inflammatory response. Concentration of IL-8 in secretions correlates with the severity of symptoms (42).

#### Immune Response: T Cells and Antibodies

HRV antigens are recognized by T cells, initiating cytotoxic T-cell responses and activating T-helper cells that drive humoral responses. A rapid expansion of epitope-specific memory T cells occurs after HRV infection allowing T-cell recruitment and facilitating viral clearance (43).

Antibodies produced during HRV infection are serotype specific. This implies the possibility of multiple HRV infections, since more than 100 different serotypes have been described to date (44).

### Virulence Factors

#### Virus-Related Virulence Factors

Severity is not related to viral copy number but seems to vary according to the serotype.

Compared to the other groups, HRV-B-related infection is less frequent and severe, causing fewer hospitalizations (45). Regarding HRV-A and HRV-C, conflicting data exist depending on studies and countries (45, 46).

#### Host Factors Related to HRV Infection or Clinical Severity

- Asthma

Immune response to HRV infection in general population is Th1 mediated. During HRV-driven asthma exacerbations, an amplified Th2 immune response is involved in IL-4, IL-5, and IL-13 productions (23, 47).

It was suggested that impaired Th1 responses could lead to poor IFN production and a lack of immune responses during HRV infection, since some authors have described a deficiency in TLR3 and melanoma differentiation-associated protein 5 (MDA-5) signaling in children with asthma (48).

- Immunocompromised Patients

In a study conducted among onco-hematological patients, despite higher viral loads and longer HRV excretion in hematopoietic cells transplants, frequency and severity of HRV infection were similar in pediatric immunocompetent and immunocompromised populations (49) and predisposed to bacterial infection of lower airways in same frequencies as other common viruses (25%) (50).

Data among non-hematological immunocompromised population are lacking to date.

- Role of MDA-5 Mutation

MDA-5 and retinoic acid inducible protein I (RIG-I) are intracellular RNA helicases induced by TLR3. They are required for effective innate immune response by their role in IFNβ expression (51).

Recent studies reported that non-functional MDA-5 leads to recurrent HRV infections and severe presentations (52, 53). That deficiency leads to a primary immunodeficiency and causes susceptibility to severe infection only with respiratory viruses as HRV.

### Secondary Bacterial Infections

Temporal correlations between HRV infection and *Streptococcus pneumoniae* (54) or *Kingella kingae* (55) have been described. Moreover, HRV infection predisposes to *S. pneumoniae* infection by overexpression of platelet-activating factor (PAF) receptor and activation of NF-kB increasing *S. pneumoniae* adherence to the airway epithelial cells (54).

In addition, HRV predisposes to S*taphylococcus aureus* infections by enhancing its internalization into respiratory epithelial cells (56). This effect does not seem to require active viral replication and seemingly involves the release of inflammatory cytokines and the overexpression of ICAM-1.

Finally, HRV can also increase paracellular permeability of airway epithelial cells after infection and even causes the loss of zona occludens 1 from tight junction complexes, disrupting airway epithelial barrier function and contributing to bacterial transmigration across the epithelial barrier and infections (57).

## Treatment

To date there is no specific antiviral treatment, and thus, HRV infection management is reduced to supportive care, rest, and hydration. Several decades ago, various antiviral molecules have been tested, but due to the traditionally expected low severity of HRV infection and limited efficiency, none have been commercialized. Zinc or vitamin C treatments did not prove clinical efficacy (58) despite *in vitro* effect (59). Steroid did not show any effect by intranasal treatment (60), but, if not showing any impact of development of asthma, systemic steroids seem to reduce recurrence in the year following first hospitalization of HRV bronchiolitis in infants aged 3–35 months (61). Finally, capsid-binding agents (62), protease 3C inhibitor (63), soluble recombinant ICAM-1 (64), and, recently, protein kinase D inhibitors (65) were tested but were not developed further, mostly because of important side effects.

Vaccination against HRV is not effective due to the large number of antigenically distinct serotypes. Nevertheless, some new ways are being explored as the use of complete VP1 protein, which could confer cross-neutralization of HRV strains (66). Thus, to date, HRV infection prevention is reduced to hand hygiene, and no specific isolation is recommended despite the increasingly pointed out HRV impacts.

## Discussion

Although HRV is commonly associated with common cold, the recent development of molecular methods has revealed that HRV is also involved in severe respiratory diseases. These new methods, such as fully automatized mPCR, have increased knowledge about the clinical manifestations of HRV by detecting it in non-respiratory diseases such as gastroenteritis or into cerebrospinal fluid.

However, involvement of HRV in the pathogenesis may be difficult to determinate in certain situations. First, asymptomatic carriage can be detected in children, either several days after a respiratory infection or in healthy children, and this carriage can reach 14–22% in children (12, 13). Second, HRV are often detected with other viruses or bacteria, especially in respiratory diseases (32) or AOM (16). Thus, although respiratory multiplex PCR was presented as an interesting diagnostic tool to reduce antibiotic prescriptions in children, the high detection rate of HRV makes difficult the interpretation of the results. Actually, the detection of HRV in <3-month-old infants (31) or during a severe pneumonia (24) does not allow to avoid antibiotic therapy due to the high risk to miss an associated bacterial infection. The generalization of multiplex PCR in hospitals and community will increase knowledge about HRV epidemiology in children, especially in non-severe presentations.

Recently, pathophysiology of HRV infections has been better investigated, and several new molecular mechanisms have been identified. Thus, some host factors have been identified to explain the ability of HRV to develop severe disease in children, such as the amplified Th2 immune response during asthma exacerbations (23, 47) or the role of MDA-5 deficiency for IFNβ production (52, 53), two essential ways for appropriate immune response. Moreover, although association between bacterial infection and viruses is known for *S. aureus* (56) or *S. pneumoniae* (54) for several years, recent data have explored the relation between *K. kingae* and HRV (55) by showing that a temporal relation exists and could be the trigger of *K. kingae* infection, suggesting that HRV infection may facilitate other bacterial infections. In the future, those results should allow describing the inflammatory response to HRV infections, the facilitating role of HRV in some bacterial invasive infections, and potentially identifying new specific immune deficiencies.

To date, neither pharmacological treatments nor vaccine is efficient, and only supportive care are recommended.

However, new treatments are studied in specific population such as HRV-induced asthma. Thus, Omalizumab, an anti-IgE therapy, has shown promising results as a preventive treatment to decrease frequency and severity of HRV exacerbations (67). Preventive treatment is also a clue for HRV control, and many researches are in progress (66). Because no preventive drug treatment exists, non-pharmaceutical interventions (washing hands, social distancing…) are still the better way to decrease the burden of HRV infections in the community (1) and control this neglected, but true, pathogen.

## Author Contributions

EB contributed to the acquisition, analysis and interpretation of the data, and drafted the article. AB, MC, FC, SL, JL, MP, PS, ES, and SW collected the data and contributed to the analysis and interpretation of data and revised the manuscript critically for important intellectual content. BV contributed to analysis and interpretation of data and revised the manuscript critically for important intellectual content. RB conceptualized and designed the study, contributed to analysis and interpretation of data, and revised the manuscript critically for important intellectual content. All authors final approved the version to be submitted.

## Conflict of Interest

The authors declare that the research was conducted in the absence of any commercial or financial relationships that could be construed as a potential conflict of interest.
